# Inter­action between maleic acid and *N*-*R*-furfuryl­amines: crystal structure of 2-methyl-*N*-[(5-phenyl­furan-2-yl)meth­yl]propan-2-aminium (2*Z*)-3-carb­oxy­acrylate and *N*-[(5-iodo­furan-2-yl)meth­yl]-2-methyl­propan-2-aminium (2*Z*)-3-carb­oxy­prop-2-enoate

**DOI:** 10.1107/S2056989017003541

**Published:** 2017-03-14

**Authors:** Elisaveta A. Kvyatkovskaya, Vladimir P. Zaytsev, Fedor I. Zubkov, Pavel V. Dorovatovskii, Yan V. Zubavichus, Victor N. Khrustalev

**Affiliations:** a Organic Chemistry Department, Peoples’ Friendship University of Russia (RUDN University), 6 Miklukho-Maklay St., Moscow 117198, Russian Federation; b National Research Centre "Kurchatov Institute", 1 Acad. Kurchatov Sq., Moscow 123182, Russian Federation; c Inorganic Chemistry Department, Peoples’ Friendship University of Russia (RUDN University), 6 Miklukho-Maklay St., Moscow 117198, Russian Federation; d X-Ray Structural Centre, A.N. Nesmeyanov Institute of Organoelement Compounds, Russian Academy of Sciences, 28 Vavilov St., B–334, Moscow 119991, Russian Federation

**Keywords:** furans, Diels–Alder reaction, maleates, crystal structure, synchrotron radiation

## Abstract

The title mol­ecular salts, C_15_H_20_NO^+^·C_4_H_3_O_4_^−^, (I), and C_9_H_15_INO^+^·C_4_H_3_O_4_^−^, (II), have very similar mol­ecular geometries for both cation and anion. The anions of both (I) and (II) are practically planar (r.m.s. deviations = 0.062 and 0.072 Å, respectively) and adopt a rare symmetrical geometry with the hy­droxy H atom approximately equidistant from the two O atoms. In their crystals, the cations and anions in both (I) and (II) form tight ionic pairs *via* strong N—H⋯O hydrogen bonds, with a roughly perpendicular disposition of the anion to the furan ring of the cation. This ion-pair conformation appears to correlate with the lack of reactivity of these salts in [4 + 2] cyclo­addition reactions. In the extended structures of (I) and (II), the ion pairs form hydrogen-bonded chains propagating along [010] and [001], respectively, *via* N—H⋯O hydrogen bonds.

## Abstract 2

The mol­ecular and crystal structures of two *N*-(furylmeth­yl)propan-2-aminium salts – the products of inter­action between maleic acid and *N*-*R*-furfuryl­amines – were studied by X-ray diffraction and correlated with their lack of reactivity in [4 + 2] cyclo­addition reactions.

## Chemical context   

Owing to the fact that the furan ring contains a system of conjugated double bonds, it usually acts as an effective diene in intra- and inter­molecular Diels–Alder reactions with electron-deficient dienophiles. The [4 + 2] cyclo­addition of furans with maleic acid leading to structurally diverse 7-oxabi­cyclo[2.2.1]heptenes has been investigated for a long time (Diels & Alder, 1931 ▸; Berson & Swidler, 1953 ▸, 1954 ▸; Eggelte *et al.*, 1973 ▸; Sprague *et al.*, 1985 ▸). However, there are only fragmentary data concerning the reactions of halogen- or aryl-substituted furans with maleic acid (Sheinkman *et al.*, 1972 ▸; Shih *et al.*, 1975 ▸). It is known that the inter­action between maleic acid and furfuryl­amines leads usually to the formation of the salts, but is not accompanied by the [4 + 2] cyclo­addition (Clitherow, 1983 ▸; Price *et al.*, 1985 ▸; Brown, 1986 ▸; Pelosi *et al.*, 2002 ▸; Craig *et al.*, 2008 ▸; Metsger *et al.*, 2010 ▸).

**Figure 1:**
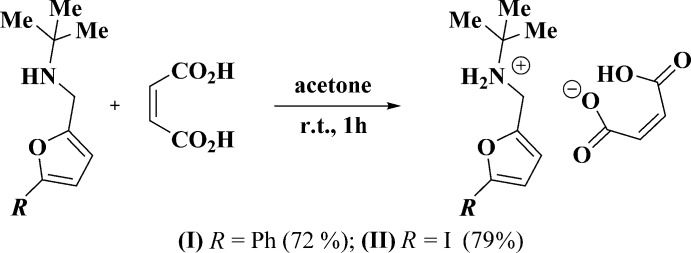
Synthesis of maleic salts (I) and (II) from *N*-[(5-*R*-furan-2-yl)meth­yl]-2-methyl­propan-2-amines.

**Figure 2:**
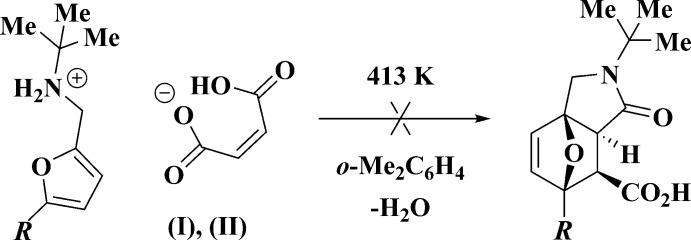
The attempted thermal cyclization of salts (I) (*R* = Ph) and (II) (*R* = I).

The main goal of this work was to study the cyclo­addition reaction between 5-*R*-furfuryl-*tert*-butyl­amines and maleic acid. The inter­action between the corresponding amines and maleic acid at room temperature leads to the salts (I) and (II) only (Fig. 1 ▸). Unexpectedly, attempts to achieve thermal cyclization of salts (I) and (II) did not result in isolation of the targeted 7-oxabi­cyclo­[2.2.1]heptenes: the initial maleates remained unchanged at temperatures up to 413 K (Fig. 2 ▸). In order to explain this fact by an understanding of their stereochemical features, an X-ray diffraction study of compounds (I) and (II) was undertaken.
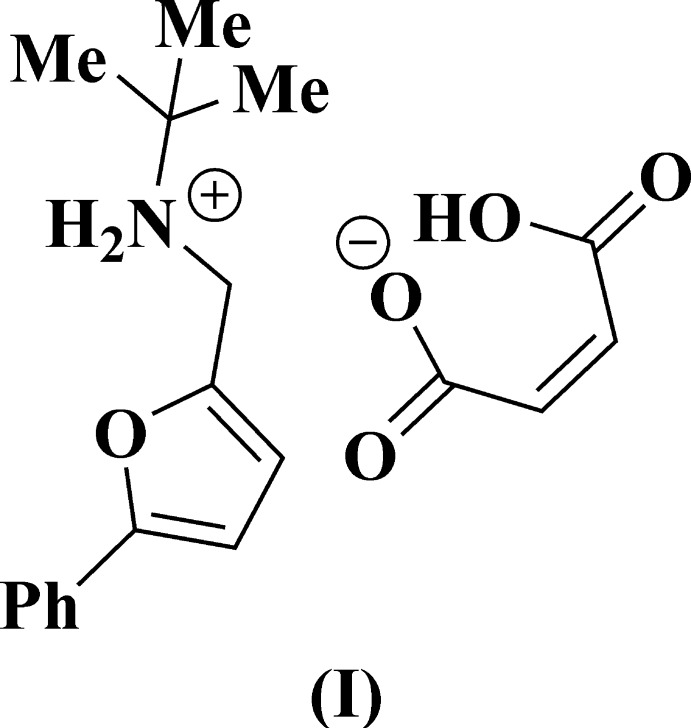
 
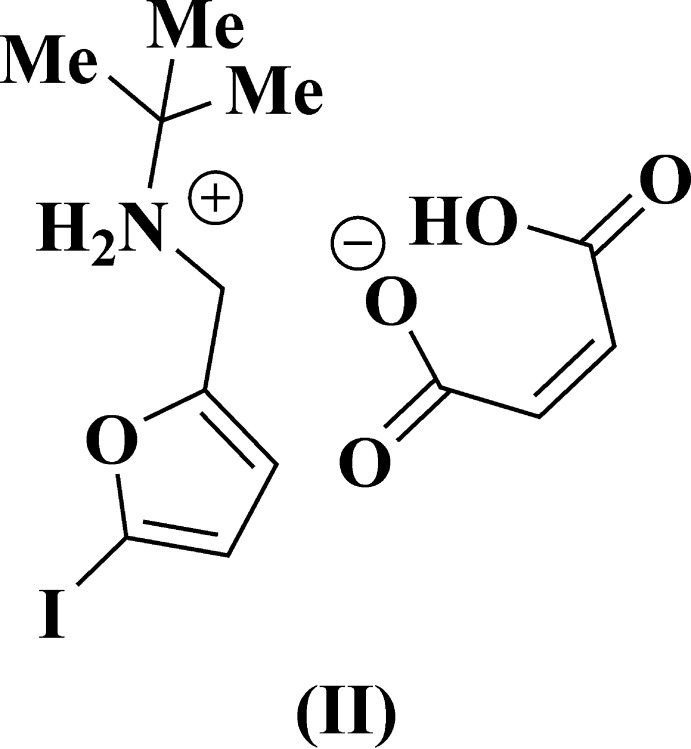


## Structural commentary   

**Figure 3:**
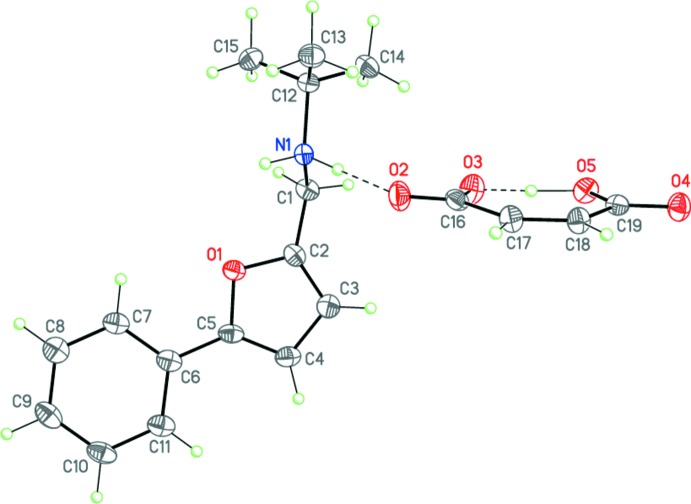
The mol­ecular structure of salt (I). Displacement ellipsoids are shown at the 50% probability level. H atoms are presented as small spheres of arbitrary radius. Dashed lines indicate the intra­molecular O—H⋯O and inter­molecular N—H⋯O hydrogen bonds.

Compounds (I), C_15_H_20_NO^+^·C_4_H_3_O_4_^−^, and (II), C_9_H_15_INO^+^·C_4_H_3_O_4_^−^, represent secondary amine salts of maleic acid and have very similar mol­ecular geometries (Figs. 3 ▸ and 4 ▸) for both cation and anion. The saturated C2–C1–N1–C(*t*-Bu) backbone of the ammonium cation is twisted by 72.66 (7) and 63.2 (2)° relative to the furan ring in (I) and (II), respectively. The phenyl substituent of the cation in (I) is almost coplanar to the furan ring (r.m.s. deviation is 0.006 Å). The anions of (I) and (II) are practically planar (r.m.s. deviations are 0.062 and 0.072 Å, respectively). It inter­esting to note that the hydrogen atom of the hy­droxy group of the anion is arranged at almost equal distances from the two oxygen atoms in both (I) and (II) (Tables 1 ▸ and 2 ▸, Figs. 3 ▸ and 4 ▸). Thus, the anions of (I) and (II) adopt a rare symmetrical geometry.

Importantly, the cations and anions in both (I) and (II) form tight ion pairs *via* strong N1—H1*A*⋯O2 hydrogen bonds (Tables 1 ▸ and 2 ▸, Figs. 3 ▸ and 4 ▸). Within the tight ion pairs, the anion is roughly perpendicular to the furan ring of the cation, the inter­planar angles being 72.01 (4) and 67.94 (12)° in (I) and (II), respectively. Apparently, the formation of the robust tight ion pairs with a definite cation–anion conformation inhibits the desired cyclization reaction, preventing the closure of the cations and anions.

## Supra­molecular features   

**Figure 5:**
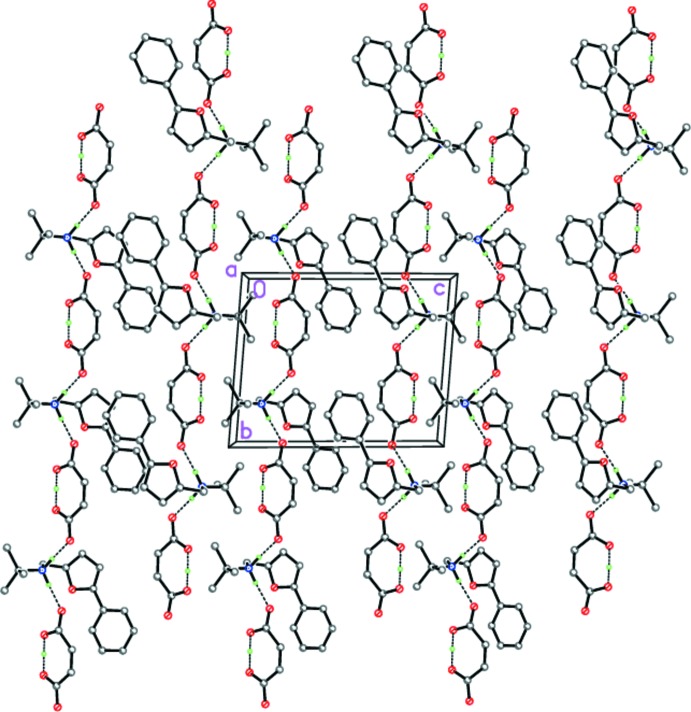
The crystal structure of (I), illustrating the hydrogen-bonded chains propagating along [010]. Dashed lines indicate the intra­molecular O—H⋯O and inter­molecular N—H⋯O hydrogen bonds.

**Figure 6:**
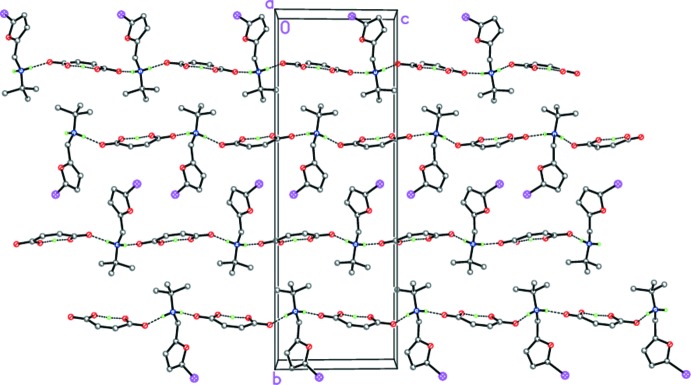
The crystal structure of (II), illustrating the hydrogen-bonded chains propagating along [001]. Dashed lines indicate the intra­molecular O—H⋯O and inter­molecular N—H⋯O hydrogen bonds.

Despite the sterically different substituents at the furyl ring of the aminium cations, compounds (I) and (II) organize similar supra­molecular structures in the solid state. So, in the crystal of (I), the tight ion pairs form hydrogen-bonded chains propagating along [010] *via* strong N1—H1*B*⋯O4 links (Table 1 ▸, Fig. 5 ▸). In the crystal of (II), the analogous hydrogen-bonded chains propagate along [001] (Table 2 ▸, Fig. 6 ▸). In both (I) and (II), the chains are further packed in stacks along [100] (Figs. 5 ▸ and 6 ▸).

## Synthesis and crystallization   

The starting *N*-[(5-R-furan-2-yl)meth­yl]-2-methyl­propan-2-amines were synthesized according to the procedure described recently (Zubkov *et al.*, 2016 ▸).

**General procedure.** A solution of the corresponding amine (1 mmol) and maleic acid (0.12 g, 1.1 mmol) in acetone (5 ml) was stirred for 1 h. The precipitated crystals were filtered off and recrystallized from an *i*-PrOH–DMF mixture [for (I)] or MeOH [for (II)] to give the analytically pure maleates (I) and (II).

**2-Methyl-** ***N*** **-[(5-phenyl­furan-2-yl)meth­yl]propan-2-amin­ium (2** ***Z*** **)-3-carb­oxy­acrylate (I)**. Colourless prisms. Yield 0.26 g (72%). M.p. = 485.1–486.1 K (*i*-PrOH–DMF). IR (KBr), ν (cm^−1^): 1591, 1630, 3435. ^1^H NMR (DMSO, 600 MHz, 301 K): δ = 1.36 (*s*, 9H, *t-*Bu), 4.30 (*s*, 2H, CH_2_—N), 6.04 (*s*, 2H, –CH=CH–), 6.74 (*d*, 1H, H3, furyl, *J* = 3.4), 7.00 (*d*, 1H, H4, furyl, *J* = 3.4), 7.34 (*br t*, 1H, H4, Ph, *J* = 7.6), 7.46 (*ddd*, 2H, H3 and H5, Ph, *J* = 8.2, *J* = 7.6, *J* = 1.4), 7.76 (*dd*, 2H, H2 and H6, Ph, *J* = 8.2, *J* = 1.4), 8.89 (*br s*, 1H, CO_2_H). ^13^C NMR (CDCl_3_, 150.9 MHz, 301 K): δ = 25.7 (3C, CH_3_), 38.0 (CH_2_—N), 57.3 (N—C), 100.0 (2C, –CH=CH–), 107.4 (C4, fur­yl), 114.3 (C3, fur­yl), 124.2, 128.5, 129.5, 130.3, 136.7 (C1, Ph), 146.6 (C2, fur­yl), 154.5 (C5, fur­yl), 167.8 (2C, CO_2_). MS (APCI): *m*/*z* = 230 [*M* − 115]^+^.

***N*** **-[(5-Iodo­furan-2-yl)meth­yl]-2-methyl­propan-2-aminium (2** ***Z*** **)-3-carb­oxy­prop-2-enoate (II)**. Colourless needles. Yield 0.31 g (79%). M.p. = 452.1–453.3 K (CH_3_OH). IR (KBr), ν (cm^−1^): 1576, 1631, 2800, 3012. ^1^H NMR (DMSO, 600 MHz, 301 K): δ = 1.26 (*s*, 9H, *t-*Bu), 4.19 (*s*, 2H, CH_2_—N), 5.99–6.00 (*m*, 2H, –CH=CH–), 6.54 (*d*, 1H, H3, furyl, *J* = 3.3), 6.73 (*d*, 1H, H4, furyl, *J* = 3.3), 8.89 (*br s*, 1H, CO_2_H). ^13^C NMR (CDCl_3_, 150.9 MHz, 301 K): δ = 25.6 (3C, CH_3_), 37.4 (CH_2_—N), 57.3 (N—C), 100.0 (C5, fur­yl), 115.3 (C4, fur­yl), 121.8 (C3, fur­yl), 136.6 (2C, —CH=CH—), 151.1 [C2, fur­yl], 167.7 (2C, CO_2_). MS (APCI): *m*/*z* = 280 [*M* − 115]^+^.

## Refinement   

Crystal data, data collection and structure refinement details are summarized in Table 3 ▸. X-ray diffraction studies for (II) were carried out on the ‘Belok’ beamline of the National Research Center "Kurchatov Institute" (Moscow, Russian Federation).

The hydrogen atoms of the amino and hy­droxy groups were localized in a difference-Fourier map and refined isotropically with fixed displacement parameters [*U*_iso_(H) = 1.2*U*_eq_(N) and 1.5*U*_eq_(O)]. All other hydrogen atoms were placed in calculated positions with C—H = 0.95–0.99 Å and refined using the riding model with fixed isotropic displacement parameters [*U*_iso_(H) = 1.5*U*_eq_(C) for the CH_3_ groups and 1.2*U*_eq_(C) for all other atoms].

## Supplementary Material

Crystal structure: contains datablock(s) global, I, II. DOI: 10.1107/S2056989017003541/hb7663sup1.cif

Structure factors: contains datablock(s) I. DOI: 10.1107/S2056989017003541/hb7663Isup2.hkl

Structure factors: contains datablock(s) II. DOI: 10.1107/S2056989017003541/hb7663IIsup3.hkl

CCDC references: 1536143, 1023931

Additional supporting information:  crystallographic information; 3D view; checkCIF report

## Figures and Tables

**Figure 4:**
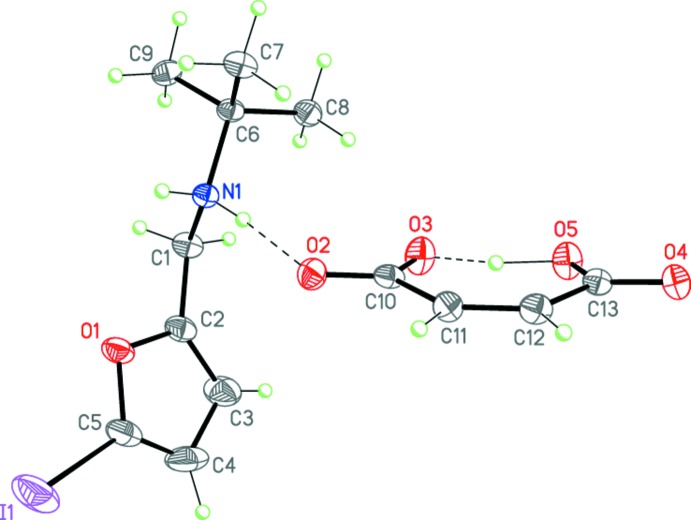
The mol­ecular structure of salt (II). Displacement ellipsoids are shown at the 50% probability level. H atoms are presented as small spheres of arbitrary radius. Dashed lines indicate the intra­molecular O—H⋯O and inter­molecular N—H⋯O hydrogen bonds.

**Table 1:** Hydrogen-bond geometry (Å, °) for (I)

*D*—H⋯*A*	*D*—H	H⋯*A*	*D*⋯*A*	*D*—H⋯*A*
O5—H5*O*⋯O3	1.160 (17)	1.257 (17)	2.4142 (14)	175.3 (15)
N1—H1*A*⋯O2^i^	0.968 (15)	1.790 (15)	2.7547 (15)	174.9 (13)
N1—H1*B*⋯O4^ii^	0.936 (15)	1.860 (15)	2.7803 (14)	167.4 (13)

Symmetry codes: (i) 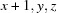; (ii) 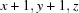.

**Table 2:** Hydrogen-bond geometry (Å, °) for (II)

*D*—H⋯*A*	*D*—H	H⋯*A*	*D*⋯*A*	*D*—H⋯*A*
O5—H5*O*⋯O3	1.18 (5)	1.25 (5)	2.425 (3)	172 (4)
N1—H1*A*⋯O2	0.88 (4)	1.97 (4)	2.828 (3)	167 (3)
N1—H1*B*⋯O4^i^	0.88 (4)	1.92 (4)	2.792 (4)	172 (3)

Symmetry code: (i) 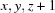.

**Table 3:** Experimental details

	(I)	(II)
Crystal data		
Chemical formula	C_15_H_20_NO^+^·C_4_H_3_O_4_^−^	C_9_H_15_INO^+^·C_4_H_3_O_4_^−^
*M*_r_	345.38	395.18
Crystal system, space group	Triclinic, *P* 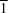	Monoclinic, *P*2_1_/*n*
Temperature (K)	120	100
*a*, *b*, *c* (Å)	7.5177 (4), 9.8339 (6), 12.1951 (7)	5.7501 (12), 28.272 (6), 9.6402 (19)
α, β, γ (°)	94.387 (1), 94.552 (1), 91.578 (1)	90, 93.17 (3), 90
*V* (Å^3^)	895.57 (9)	1564.8 (6)
*Z*	2	4
Radiation type	Mo *K*α	Synchrotron, λ = 0.96990 Å
μ (mm^−1^)	0.09	4.69
Crystal size (mm)	0.30 × 0.25 × 0.20	0.30 × 0.05 × 0.03

Data collection		
Diffractometer	Bruker APEXII CCD	Rayonix SX165 CCD
Absorption correction	Multi-scan (*SADABS*; Sheldrick, 2003 ▸)	Multi-scan (*SCALA*; Evans, 2006 ▸)
*T*_min_, *T*_max_	0.966, 0.977	0.460, 0.860
No. of measured, independent and observed [*I* > 2σ(*I*)] reflections	14165, 6555, 3947	21875, 3146, 2714
*R*_int_	0.047	0.068
(sin θ/λ)_max_ (Å^−1^)	0.760	0.641

Refinement		
*R*[*F* ^2^ > 2σ(*F* ^2^)], *wR*(*F* ^2^), *S*	0.053, 0.122, 1.00	0.040, 0.100, 1.02
No. of reflections	6555	3146
No. of parameters	238	194
H-atom treatment	H atoms treated by a mixture of independent and constrained refinement	H atoms treated by a mixture of independent and constrained refinement
Δρ_max_, Δρ_min_ (e Å^−3^)	0.31, −0.26	0.94, −1.21

Computer programs: *APEX2* (Bruker, 2005 ▸), *SAINT* (Bruker, 2001 ▸), *Automar* (MarXperts, 2015 ▸), *iMosflm* (Battye *et al.*, 2011 ▸), *SHELXT* (Sheldrick, 2015*a* ▸), *SHELXL2014* (Sheldrick, 2015*b* ▸) and *SHELXTL* (Sheldrick, 2008 ▸).

## supplementary crystallographic information

### (I) 2-Methyl-N-[(5-phenylfuran-2-yl)methyl]propan-2-aminium (2Z)-3-carboxyacrylate Crystal data

**Table:**

C_15_H_20_NO^+^·C_4_H_3_O_4_^−^	*Z* = 2
*M**_r_* = 345.38	*F*(000) = 368
Triclinic, *P*1	*D*_x_ = 1.281 Mg m^−^^3^
*a* = 7.5177 (4) Å	Mo *K*α radiation, λ = 0.71073 Å
*b* = 9.8339 (6) Å	Cell parameters from 2186 reflections
*c* = 12.1951 (7) Å	θ = 2.6–31.5°
α = 94.387 (1)°	µ = 0.09 mm^−^^1^
β = 94.552 (1)°	*T* = 120 K
γ = 91.578 (1)°	Prism, colourless
*V* = 895.57 (9) Å^3^	0.30 × 0.25 × 0.20 mm

### (I) 2-Methyl-N-[(5-phenylfuran-2-yl)methyl]propan-2-aminium (2Z)-3-carboxyacrylate Data collection

**Table:**

Bruker APEXII CCD diffractometer	3947 reflections with *I* > 2σ(*I*)
Radiation source: fine-focus sealed tube	*R*_int_ = 0.047
φ and ω scans	θ_max_ = 32.7°, θ_min_ = 2.1°
Absorption correction: multi-scan (SADABS; Sheldrick, 2003)	*h* = −11→11
*T*_min_ = 0.966, *T*_max_ = 0.977	*k* = −14→14
14165 measured reflections	*l* = −18→18
6555 independent reflections	

### (I) 2-Methyl-N-[(5-phenylfuran-2-yl)methyl]propan-2-aminium (2Z)-3-carboxyacrylate Refinement

**Table:**

Refinement on *F*^2^	Primary atom site location: difference Fourier map
Least-squares matrix: full	Secondary atom site location: difference Fourier map
*R*[*F*^2^ > 2σ(*F*^2^)] = 0.053	Hydrogen site location: mixed
*wR*(*F*^2^) = 0.122	H atoms treated by a mixture of independent and constrained refinement
*S* = 1.00	*w* = 1/[σ^2^(*F*_o_^2^) + (0.0454*P*)^2^] where *P* = (*F*_o_^2^ + 2*F*_c_^2^)/3
6555 reflections	(Δ/σ)_max_ < 0.001
238 parameters	Δρ_max_ = 0.31 e Å^−^^3^
0 restraints	Δρ_min_ = −0.26 e Å^−^^3^

### (I) 2-Methyl-N-[(5-phenylfuran-2-yl)methyl]propan-2-aminium (2Z)-3-carboxyacrylate Special details

**Table:**

Geometry. All esds (except the esd in the dihedral angle between two l.s. planes) are estimated using the full covariance matrix. The cell esds are taken into account individually in the estimation of esds in distances, angles and torsion angles; correlations between esds in cell parameters are only used when they are defined by crystal symmetry. An approximate (isotropic) treatment of cell esds is used for estimating esds involving l.s. planes.

### (I) 2-Methyl-N-[(5-phenylfuran-2-yl)methyl]propan-2-aminium (2Z)-3-carboxyacrylate Fractional atomic coordinates and isotropic or equivalent isotropic displacement parameters (Å^2^)

**Table:**

	*x*	*y*	*z*	*U*_iso_*/*U*_eq_	
O1	0.92461 (12)	0.90909 (9)	0.24482 (7)	0.0223 (2)	
N1	1.20595 (14)	0.76402 (11)	0.11610 (9)	0.0175 (2)	
H1A	1.2498 (18)	0.6949 (15)	0.1627 (12)	0.021*	
H1B	1.2370 (19)	0.8484 (15)	0.1545 (12)	0.021*	
C1	1.00641 (17)	0.74679 (14)	0.09978 (11)	0.0223 (3)	
H1C	0.9596	0.8102	0.0461	0.027*	
H1D	0.9736	0.6525	0.0691	0.027*	
C2	0.92419 (17)	0.77432 (13)	0.20536 (11)	0.0216 (3)	
C3	0.84120 (18)	0.69612 (15)	0.27361 (12)	0.0264 (3)	
H3	0.8233	0.5998	0.2652	0.032*	
C4	0.78576 (19)	0.78600 (15)	0.36059 (12)	0.0272 (3)	
H4	0.7229	0.7610	0.4210	0.033*	
C5	0.83939 (17)	0.91370 (14)	0.34107 (11)	0.0222 (3)	
C6	0.82740 (17)	1.04880 (14)	0.39825 (11)	0.0229 (3)	
C7	0.89734 (18)	1.16539 (15)	0.35647 (12)	0.0262 (3)	
H7	0.9550	1.1572	0.2898	0.031*	
C8	0.88348 (19)	1.29304 (16)	0.41130 (12)	0.0296 (3)	
H8	0.9325	1.3716	0.3824	0.036*	
C9	0.79823 (19)	1.30640 (16)	0.50820 (12)	0.0302 (3)	
H9	0.7881	1.3940	0.5454	0.036*	
C10	0.72778 (19)	1.19148 (16)	0.55059 (11)	0.0286 (3)	
H10	0.6689	1.2005	0.6167	0.034*	
C11	0.74308 (18)	1.06376 (16)	0.49678 (11)	0.0260 (3)	
H11	0.6960	0.9854	0.5269	0.031*	
C12	1.30888 (17)	0.75667 (13)	0.01359 (11)	0.0200 (3)	
C13	1.50545 (18)	0.75962 (15)	0.05686 (12)	0.0265 (3)	
H13A	1.5326	0.8418	0.1067	0.040*	
H13B	1.5804	0.7604	−0.0053	0.040*	
H13C	1.5294	0.6785	0.0969	0.040*	
C14	1.25942 (19)	0.62298 (14)	−0.05538 (12)	0.0262 (3)	
H14A	1.1346	0.6240	−0.0853	0.039*	
H14B	1.2751	0.5465	−0.0089	0.039*	
H14C	1.3368	0.6126	−0.1163	0.039*	
C15	1.26620 (19)	0.88007 (15)	−0.05105 (12)	0.0262 (3)	
H15A	1.2918	0.9639	−0.0029	0.039*	
H15B	1.1397	0.8753	−0.0779	0.039*	
H15C	1.3397	0.8804	−0.1139	0.039*	
O2	0.34880 (14)	0.57638 (10)	0.25174 (8)	0.0310 (2)	
O3	0.19310 (13)	0.40290 (11)	0.15970 (8)	0.0306 (2)	
O4	0.34720 (13)	−0.00091 (10)	0.23666 (8)	0.0279 (2)	
O5	0.19913 (12)	0.15695 (10)	0.14960 (8)	0.0255 (2)	
H5O	0.190 (2)	0.2747 (18)	0.1531 (13)	0.038*	
C16	0.31067 (18)	0.45314 (14)	0.23545 (11)	0.0226 (3)	
C17	0.40964 (18)	0.36056 (14)	0.30859 (11)	0.0235 (3)	
H17	0.4830	0.4061	0.3680	0.028*	
C18	0.41217 (18)	0.22465 (14)	0.30440 (11)	0.0226 (3)	
H18	0.4888	0.1892	0.3602	0.027*	
C19	0.31356 (17)	0.11924 (14)	0.22580 (11)	0.0210 (3)	

### (I) 2-Methyl-N-[(5-phenylfuran-2-yl)methyl]propan-2-aminium (2Z)-3-carboxyacrylate Atomic displacement parameters (Å^2^)

**Table:**

	*U*^11^	*U*^22^	*U*^33^	*U*^12^	*U*^13^	*U*^23^
O1	0.0221 (5)	0.0253 (5)	0.0199 (5)	0.0034 (4)	0.0041 (4)	0.0016 (4)
N1	0.0174 (5)	0.0160 (5)	0.0189 (5)	0.0008 (4)	−0.0002 (4)	0.0010 (4)
C1	0.0167 (6)	0.0276 (7)	0.0217 (6)	0.0006 (5)	−0.0003 (5)	−0.0016 (5)
C2	0.0178 (6)	0.0236 (7)	0.0225 (6)	0.0032 (5)	−0.0012 (5)	−0.0011 (5)
C3	0.0246 (7)	0.0266 (7)	0.0281 (7)	0.0009 (6)	0.0022 (6)	0.0025 (6)
C4	0.0254 (7)	0.0333 (8)	0.0242 (7)	0.0024 (6)	0.0068 (6)	0.0056 (6)
C5	0.0181 (6)	0.0320 (7)	0.0172 (6)	0.0057 (5)	0.0023 (5)	0.0039 (5)
C6	0.0183 (6)	0.0309 (7)	0.0197 (6)	0.0072 (5)	−0.0001 (5)	0.0022 (5)
C7	0.0242 (7)	0.0325 (8)	0.0223 (7)	0.0043 (6)	0.0039 (5)	0.0022 (6)
C8	0.0276 (7)	0.0318 (8)	0.0293 (8)	0.0040 (6)	0.0008 (6)	0.0020 (6)
C9	0.0289 (7)	0.0348 (8)	0.0252 (7)	0.0090 (6)	−0.0028 (6)	−0.0064 (6)
C10	0.0252 (7)	0.0417 (9)	0.0189 (7)	0.0105 (6)	0.0005 (5)	−0.0004 (6)
C11	0.0227 (7)	0.0359 (8)	0.0197 (7)	0.0077 (6)	0.0006 (5)	0.0038 (6)
C12	0.0189 (6)	0.0220 (6)	0.0193 (6)	0.0012 (5)	0.0031 (5)	0.0007 (5)
C13	0.0207 (6)	0.0299 (8)	0.0291 (7)	0.0013 (6)	0.0037 (6)	0.0026 (6)
C14	0.0260 (7)	0.0261 (7)	0.0257 (7)	0.0021 (6)	0.0042 (6)	−0.0051 (6)
C15	0.0287 (7)	0.0276 (7)	0.0229 (7)	0.0005 (6)	0.0025 (6)	0.0062 (5)
O2	0.0433 (6)	0.0204 (5)	0.0294 (6)	0.0081 (4)	0.0000 (5)	0.0026 (4)
O3	0.0296 (5)	0.0296 (6)	0.0318 (6)	0.0042 (4)	−0.0079 (4)	0.0075 (4)
O4	0.0333 (6)	0.0196 (5)	0.0301 (5)	−0.0014 (4)	−0.0006 (4)	−0.0001 (4)
O5	0.0256 (5)	0.0284 (5)	0.0215 (5)	−0.0018 (4)	−0.0041 (4)	0.0021 (4)
C16	0.0233 (6)	0.0250 (7)	0.0203 (6)	0.0077 (5)	0.0039 (5)	0.0025 (5)
C17	0.0254 (7)	0.0222 (7)	0.0217 (7)	0.0024 (5)	−0.0045 (5)	−0.0003 (5)
C18	0.0225 (6)	0.0226 (7)	0.0218 (6)	0.0029 (5)	−0.0042 (5)	0.0021 (5)
C19	0.0206 (6)	0.0230 (7)	0.0191 (6)	−0.0007 (5)	0.0031 (5)	0.0000 (5)

### (I) 2-Methyl-N-[(5-phenylfuran-2-yl)methyl]propan-2-aminium (2Z)-3-carboxyacrylate Geometric parameters (Å, º)

**Table:**

O1—C2	1.3747 (16)	C11—H11	0.9500
O1—C5	1.3796 (15)	C12—C15	1.5247 (19)
N1—C1	1.5007 (16)	C12—C14	1.5259 (18)
N1—C12	1.5198 (16)	C12—C13	1.5279 (18)
N1—H1A	0.968 (15)	C13—H13A	0.9800
N1—H1B	0.936 (15)	C13—H13B	0.9800
C1—C2	1.4817 (18)	C13—H13C	0.9800
C1—H1C	0.9900	C14—H14A	0.9800
C1—H1D	0.9900	C14—H14B	0.9800
C2—C3	1.352 (2)	C14—H14C	0.9800
C3—C4	1.424 (2)	C15—H15A	0.9800
C3—H3	0.9500	C15—H15B	0.9800
C4—C5	1.353 (2)	C15—H15C	0.9800
C4—H4	0.9500	O2—C16	1.2351 (17)
C5—C6	1.4609 (19)	O3—C16	1.2866 (17)
C6—C7	1.396 (2)	O3—H5O	1.257 (17)
C6—C11	1.4022 (19)	O4—C19	1.2300 (16)
C7—C8	1.387 (2)	O5—C19	1.2979 (16)
C7—H7	0.9500	O5—H5O	1.160 (17)
C8—C9	1.388 (2)	C16—C17	1.4947 (19)
C8—H8	0.9500	C17—C18	1.3343 (18)
C9—C10	1.387 (2)	C17—H17	0.9500
C9—H9	0.9500	C18—C19	1.4952 (18)
C10—C11	1.384 (2)	C18—H18	0.9500
C10—H10	0.9500		
			
C2—O1—C5	106.85 (10)	C10—C11—H11	119.7
C1—N1—C12	117.49 (10)	C6—C11—H11	119.7
C1—N1—H1A	108.0 (8)	N1—C12—C15	108.92 (10)
C12—N1—H1A	108.0 (8)	N1—C12—C14	109.41 (11)
C1—N1—H1B	109.6 (9)	C15—C12—C14	111.64 (11)
C12—N1—H1B	106.5 (9)	N1—C12—C13	105.08 (10)
H1A—N1—H1B	106.6 (12)	C15—C12—C13	111.28 (11)
C2—C1—N1	111.02 (10)	C14—C12—C13	110.29 (11)
C2—C1—H1C	109.4	C12—C13—H13A	109.5
N1—C1—H1C	109.4	C12—C13—H13B	109.5
C2—C1—H1D	109.4	H13A—C13—H13B	109.5
N1—C1—H1D	109.4	C12—C13—H13C	109.5
H1C—C1—H1D	108.0	H13A—C13—H13C	109.5
C3—C2—O1	109.83 (12)	H13B—C13—H13C	109.5
C3—C2—C1	134.49 (13)	C12—C14—H14A	109.5
O1—C2—C1	115.65 (12)	C12—C14—H14B	109.5
C2—C3—C4	106.78 (13)	H14A—C14—H14B	109.5
C2—C3—H3	126.6	C12—C14—H14C	109.5
C4—C3—H3	126.6	H14A—C14—H14C	109.5
C5—C4—C3	107.10 (12)	H14B—C14—H14C	109.5
C5—C4—H4	126.4	C12—C15—H15A	109.5
C3—C4—H4	126.4	C12—C15—H15B	109.5
C4—C5—O1	109.43 (12)	H15A—C15—H15B	109.5
C4—C5—C6	134.61 (13)	C12—C15—H15C	109.5
O1—C5—C6	115.96 (12)	H15A—C15—H15C	109.5
C7—C6—C11	118.51 (13)	H15B—C15—H15C	109.5
C7—C6—C5	121.38 (12)	C16—O3—H5O	111.3 (7)
C11—C6—C5	120.11 (13)	C19—O5—H5O	111.7 (8)
C8—C7—C6	120.65 (13)	O2—C16—O3	123.38 (13)
C8—C7—H7	119.7	O2—C16—C17	116.77 (12)
C6—C7—H7	119.7	O3—C16—C17	119.85 (12)
C7—C8—C9	120.20 (15)	C18—C17—C16	130.78 (13)
C7—C8—H8	119.9	C18—C17—H17	114.6
C9—C8—H8	119.9	C16—C17—H17	114.6
C10—C9—C8	119.80 (14)	C17—C18—C19	130.28 (12)
C10—C9—H9	120.1	C17—C18—H18	114.9
C8—C9—H9	120.1	C19—C18—H18	114.9
C11—C10—C9	120.17 (13)	O4—C19—O5	123.00 (12)
C11—C10—H10	119.9	O4—C19—C18	117.33 (12)
C9—C10—H10	119.9	O5—C19—C18	119.67 (12)
C10—C11—C6	120.67 (14)		
			
C12—N1—C1—C2	−172.59 (11)	C11—C6—C7—C8	0.0 (2)
C5—O1—C2—C3	0.25 (14)	C5—C6—C7—C8	179.45 (13)
C5—O1—C2—C1	178.57 (11)	C6—C7—C8—C9	−0.6 (2)
N1—C1—C2—C3	−109.20 (17)	C7—C8—C9—C10	0.4 (2)
N1—C1—C2—O1	73.02 (14)	C8—C9—C10—C11	0.3 (2)
O1—C2—C3—C4	0.17 (15)	C9—C10—C11—C6	−0.9 (2)
C1—C2—C3—C4	−177.70 (14)	C7—C6—C11—C10	0.8 (2)
C2—C3—C4—C5	−0.53 (16)	C5—C6—C11—C10	−178.73 (12)
C3—C4—C5—O1	0.70 (16)	C1—N1—C12—C15	67.55 (14)
C3—C4—C5—C6	−178.87 (14)	C1—N1—C12—C14	−54.73 (14)
C2—O1—C5—C4	−0.60 (14)	C1—N1—C12—C13	−173.13 (11)
C2—O1—C5—C6	179.06 (11)	O2—C16—C17—C18	−172.20 (14)
C4—C5—C6—C7	−179.94 (15)	O3—C16—C17—C18	7.4 (2)
O1—C5—C6—C7	0.51 (18)	C16—C17—C18—C19	−1.3 (3)
C4—C5—C6—C11	−0.5 (2)	C17—C18—C19—O4	176.22 (14)
O1—C5—C6—C11	179.98 (11)	C17—C18—C19—O5	−3.2 (2)

### (I) 2-Methyl-N-[(5-phenylfuran-2-yl)methyl]propan-2-aminium (2Z)-3-carboxyacrylate Hydrogen-bond geometry (Å, º)

**Table:**

*D*—H···*A*	*D*—H	H···*A*	*D*···*A*	*D*—H···*A*
O5—H5*O*···O3	1.160 (17)	1.257 (17)	2.4142 (14)	175.3 (15)
N1—H1*A*···O2^i^	0.968 (15)	1.790 (15)	2.7547 (15)	174.9 (13)
N1—H1*B*···O4^ii^	0.936 (15)	1.860 (15)	2.7803 (14)	167.4 (13)

Symmetry codes: (i) *x*+1, *y*, *z*; (ii) *x*+1, *y*+1, *z*.

### (II) N-[(5-Iodofuran-2-yl)methyl]-2-methylpropan-2-aminium (2Z)-3-carboxyprop-2-enoate Crystal data

**Table:**

C_9_H_15_INO^+^·C_4_H_3_O_4_^−^	*F*(000) = 784
*M**_r_* = 395.18	*D*_x_ = 1.678 Mg m^−^^3^
Monoclinic, *P*2_1_/*n*	Synchrotron radiation, λ = 0.96990 Å
*a* = 5.7501 (12) Å	Cell parameters from 600 reflections
*b* = 28.272 (6) Å	θ = 3.5–35.0°
*c* = 9.6402 (19) Å	µ = 4.69 mm^−^^1^
β = 93.17 (3)°	*T* = 100 K
*V* = 1564.8 (6) Å^3^	Needle, colourless
*Z* = 4	0.30 × 0.05 × 0.03 mm

### (II) N-[(5-Iodofuran-2-yl)methyl]-2-methylpropan-2-aminium (2Z)-3-carboxyprop-2-enoate Data collection

**Table:**

Rayonix SX165 CCD diffractometer	2714 reflections with *I* > 2σ(*I*)
φ scan	*R*_int_ = 0.068
Absorption correction: multi-scan (*Scala*; Evans, 2006)	θ_max_ = 38.5°, θ_min_ = 3.5°
*T*_min_ = 0.460, *T*_max_ = 0.860	*h* = −7→7
21875 measured reflections	*k* = −36→36
3146 independent reflections	*l* = −11→11

### (II) N-[(5-Iodofuran-2-yl)methyl]-2-methylpropan-2-aminium (2Z)-3-carboxyprop-2-enoate Refinement

**Table:**

Refinement on *F*^2^	Secondary atom site location: difference Fourier map
Least-squares matrix: full	Hydrogen site location: mixed
*R*[*F*^2^ > 2σ(*F*^2^)] = 0.040	H atoms treated by a mixture of independent and constrained refinement
*wR*(*F*^2^) = 0.100	*w* = 1/[σ^2^(*F*_o_^2^) + (0.0377*P*)^2^ + 3.*P*] where *P* = (*F*_o_^2^ + 2*F*_c_^2^)/3
*S* = 1.02	(Δ/σ)_max_ < 0.001
3146 reflections	Δρ_max_ = 0.94 e Å^−^^3^
194 parameters	Δρ_min_ = −1.21 e Å^−^^3^
0 restraints	Extinction correction: SHELXL2014 (Sheldrick, 2015a), Fc^*^=kFc[1+0.001xFc^2^λ^3^/sin(2θ)]^-1/4^
Primary atom site location: difference Fourier map	Extinction coefficient: 0.0047 (5)

### (II) N-[(5-Iodofuran-2-yl)methyl]-2-methylpropan-2-aminium (2Z)-3-carboxyprop-2-enoate Special details

**Table:**

Geometry. All esds (except the esd in the dihedral angle between two l.s. planes) are estimated using the full covariance matrix. The cell esds are taken into account individually in the estimation of esds in distances, angles and torsion angles; correlations between esds in cell parameters are only used when they are defined by crystal symmetry. An approximate (isotropic) treatment of cell esds is used for estimating esds involving l.s. planes.

### (II) N-[(5-Iodofuran-2-yl)methyl]-2-methylpropan-2-aminium (2Z)-3-carboxyprop-2-enoate Fractional atomic coordinates and isotropic or equivalent isotropic displacement parameters (Å^2^)

**Table:**

	*x*	*y*	*z*	*U*_iso_*/*U*_eq_	
I1	0.75296 (5)	0.48487 (2)	0.86223 (3)	0.05279 (17)	
O1	0.4289 (4)	0.56119 (7)	0.7766 (3)	0.0273 (5)	
O2	0.6669 (4)	0.62755 (8)	0.4628 (2)	0.0250 (5)	
O3	0.4201 (4)	0.64214 (9)	0.2824 (3)	0.0284 (5)	
O4	0.7068 (4)	0.65296 (8)	−0.1219 (2)	0.0242 (5)	
O5	0.4335 (4)	0.65188 (8)	0.0330 (3)	0.0272 (5)	
H5O	0.434 (7)	0.6447 (14)	0.153 (5)	0.041*	
N1	0.3606 (4)	0.65833 (8)	0.6639 (3)	0.0172 (5)	
H1A	0.436 (6)	0.6486 (12)	0.592 (4)	0.021*	
H1B	0.462 (7)	0.6587 (12)	0.735 (4)	0.021*	
C1	0.1674 (5)	0.62389 (10)	0.6907 (4)	0.0227 (7)	
H1C	0.1109	0.6293	0.7845	0.027*	
H1D	0.0355	0.6289	0.6219	0.027*	
C2	0.2549 (5)	0.57433 (10)	0.6802 (4)	0.0247 (7)	
C3	0.2006 (7)	0.53816 (12)	0.5920 (5)	0.0379 (9)	
H3	0.0876	0.5385	0.5163	0.045*	
C4	0.3478 (8)	0.49932 (12)	0.6355 (5)	0.0425 (10)	
H4	0.3511	0.4687	0.5950	0.051*	
C5	0.4796 (6)	0.51500 (11)	0.7450 (4)	0.0307 (9)	
C6	0.2890 (5)	0.71010 (10)	0.6427 (3)	0.0189 (7)	
C7	0.5174 (5)	0.73740 (10)	0.6298 (4)	0.0236 (7)	
H7A	0.6151	0.7339	0.7157	0.035*	
H7B	0.4829	0.7710	0.6137	0.035*	
H7C	0.6001	0.7248	0.5516	0.035*	
C8	0.1369 (5)	0.71450 (11)	0.5089 (4)	0.0241 (7)	
H8A	0.2219	0.7025	0.4310	0.036*	
H8B	0.0968	0.7478	0.4926	0.036*	
H8C	−0.0061	0.6961	0.5171	0.036*	
C9	0.1632 (5)	0.72673 (10)	0.7693 (4)	0.0225 (7)	
H9A	0.0164	0.7094	0.7744	0.034*	
H9B	0.1308	0.7607	0.7611	0.034*	
H9C	0.2618	0.7208	0.8537	0.034*	
C10	0.6235 (5)	0.63160 (9)	0.3358 (4)	0.0198 (7)	
C11	0.8219 (5)	0.62384 (10)	0.2425 (3)	0.0213 (7)	
H11	0.9625	0.6131	0.2884	0.026*	
C12	0.8311 (5)	0.62967 (10)	0.1049 (4)	0.0225 (7)	
H12	0.9776	0.6225	0.0690	0.027*	
C13	0.6458 (5)	0.64577 (10)	−0.0025 (3)	0.0196 (6)	

### (II) N-[(5-Iodofuran-2-yl)methyl]-2-methylpropan-2-aminium (2Z)-3-carboxyprop-2-enoate Atomic displacement parameters (Å^2^)

**Table:**

	*U*^11^	*U*^22^	*U*^33^	*U*^12^	*U*^13^	*U*^23^
I1	0.0535 (2)	0.04314 (19)	0.0625 (3)	0.02765 (12)	0.00979 (16)	0.01596 (12)
O1	0.0296 (12)	0.0202 (10)	0.0322 (15)	0.0075 (9)	0.0039 (11)	0.0029 (9)
O2	0.0267 (12)	0.0268 (11)	0.0221 (14)	0.0022 (9)	0.0057 (10)	0.0000 (9)
O3	0.0182 (11)	0.0421 (13)	0.0254 (14)	0.0036 (9)	0.0057 (10)	0.0003 (10)
O4	0.0217 (11)	0.0293 (11)	0.0218 (13)	−0.0005 (8)	0.0017 (10)	0.0046 (9)
O5	0.0164 (10)	0.0397 (13)	0.0257 (14)	0.0047 (9)	0.0030 (9)	0.0035 (10)
N1	0.0150 (12)	0.0167 (11)	0.0201 (15)	0.0015 (9)	0.0032 (11)	0.0001 (10)
C1	0.0178 (14)	0.0199 (14)	0.031 (2)	−0.0007 (11)	0.0050 (13)	0.0017 (13)
C2	0.0235 (15)	0.0194 (14)	0.032 (2)	−0.0016 (11)	0.0062 (14)	0.0039 (13)
C3	0.042 (2)	0.0239 (16)	0.046 (3)	−0.0080 (14)	−0.0075 (18)	0.0007 (16)
C4	0.054 (2)	0.0180 (15)	0.056 (3)	−0.0022 (16)	0.011 (2)	−0.0060 (17)
C5	0.0343 (18)	0.0207 (15)	0.038 (2)	0.0063 (12)	0.0127 (17)	0.0074 (14)
C6	0.0180 (14)	0.0159 (13)	0.0231 (19)	0.0027 (10)	0.0033 (13)	−0.0002 (11)
C7	0.0210 (15)	0.0182 (13)	0.032 (2)	0.0006 (11)	0.0046 (14)	0.0021 (13)
C8	0.0237 (15)	0.0224 (14)	0.0260 (19)	0.0045 (11)	−0.0001 (14)	0.0007 (13)
C9	0.0198 (14)	0.0214 (13)	0.0267 (19)	0.0024 (11)	0.0046 (13)	−0.0040 (13)
C10	0.0189 (14)	0.0144 (12)	0.026 (2)	−0.0001 (10)	0.0054 (13)	0.0007 (12)
C11	0.0180 (14)	0.0229 (14)	0.0232 (19)	0.0023 (11)	0.0034 (13)	0.0016 (13)
C12	0.0164 (14)	0.0230 (14)	0.029 (2)	0.0026 (11)	0.0065 (13)	0.0003 (13)
C13	0.0172 (13)	0.0181 (13)	0.0235 (19)	−0.0005 (10)	0.0014 (13)	0.0007 (12)

### (II) N-[(5-Iodofuran-2-yl)methyl]-2-methylpropan-2-aminium (2Z)-3-carboxyprop-2-enoate Geometric parameters (Å, º)

**Table:**

I1—C5	2.068 (4)	C4—C5	1.341 (6)
O1—C5	1.376 (4)	C4—H4	0.9500
O1—C2	1.379 (4)	C6—C8	1.523 (5)
O2—C10	1.241 (4)	C6—C9	1.527 (4)
O3—C10	1.287 (4)	C6—C7	1.535 (4)
O3—H5O	1.25 (5)	C7—H7A	0.9800
O4—C13	1.238 (4)	C7—H7B	0.9800
O5—C13	1.297 (3)	C7—H7C	0.9800
O5—H5O	1.18 (5)	C8—H8A	0.9800
N1—C1	1.510 (4)	C8—H8B	0.9800
N1—C6	1.531 (4)	C8—H8C	0.9800
N1—H1A	0.88 (4)	C9—H9A	0.9800
N1—H1B	0.88 (4)	C9—H9B	0.9800
C1—C2	1.494 (4)	C9—H9C	0.9800
C1—H1C	0.9900	C10—C11	1.507 (4)
C1—H1D	0.9900	C11—C12	1.340 (5)
C2—C3	1.355 (5)	C11—H11	0.9500
C3—C4	1.435 (6)	C12—C13	1.515 (5)
C3—H3	0.9500	C12—H12	0.9500
			
C5—O1—C2	105.2 (3)	N1—C6—C7	105.5 (2)
C10—O3—H5O	107.7 (19)	C6—C7—H7A	109.5
C13—O5—H5O	107 (2)	C6—C7—H7B	109.5
C1—N1—C6	116.4 (2)	H7A—C7—H7B	109.5
C1—N1—H1A	109 (2)	C6—C7—H7C	109.5
C6—N1—H1A	109 (2)	H7A—C7—H7C	109.5
C1—N1—H1B	110 (2)	H7B—C7—H7C	109.5
C6—N1—H1B	105 (2)	C6—C8—H8A	109.5
H1A—N1—H1B	107 (3)	C6—C8—H8B	109.5
C2—C1—N1	109.8 (2)	H8A—C8—H8B	109.5
C2—C1—H1C	109.7	C6—C8—H8C	109.5
N1—C1—H1C	109.7	H8A—C8—H8C	109.5
C2—C1—H1D	109.7	H8B—C8—H8C	109.5
N1—C1—H1D	109.7	C6—C9—H9A	109.5
H1C—C1—H1D	108.2	C6—C9—H9B	109.5
C3—C2—O1	110.7 (3)	H9A—C9—H9B	109.5
C3—C2—C1	133.1 (3)	C6—C9—H9C	109.5
O1—C2—C1	116.2 (3)	H9A—C9—H9C	109.5
C2—C3—C4	106.4 (4)	H9B—C9—H9C	109.5
C2—C3—H3	126.8	O2—C10—O3	123.0 (3)
C4—C3—H3	126.8	O2—C10—C11	117.3 (3)
C5—C4—C3	106.0 (3)	O3—C10—C11	119.7 (3)
C5—C4—H4	127.0	C12—C11—C10	130.2 (3)
C3—C4—H4	127.0	C12—C11—H11	114.9
C4—C5—O1	111.8 (3)	C10—C11—H11	114.9
C4—C5—I1	132.4 (3)	C11—C12—C13	130.5 (3)
O1—C5—I1	115.7 (3)	C11—C12—H12	114.8
C8—C6—C9	112.1 (3)	C13—C12—H12	114.8
C8—C6—N1	109.2 (2)	O4—C13—O5	122.9 (3)
C9—C6—N1	108.9 (2)	O4—C13—C12	117.4 (3)
C8—C6—C7	110.1 (3)	O5—C13—C12	119.7 (3)
C9—C6—C7	110.8 (3)		
			
C6—N1—C1—C2	168.5 (3)	C2—O1—C5—C4	0.1 (4)
C5—O1—C2—C3	−0.4 (4)	C2—O1—C5—I1	175.9 (2)
C5—O1—C2—C1	−179.6 (3)	C1—N1—C6—C8	−66.1 (3)
N1—C1—C2—C3	−115.3 (4)	C1—N1—C6—C9	56.6 (4)
N1—C1—C2—O1	63.7 (4)	C1—N1—C6—C7	175.5 (3)
O1—C2—C3—C4	0.5 (4)	O2—C10—C11—C12	−173.7 (3)
C1—C2—C3—C4	179.6 (3)	O3—C10—C11—C12	6.0 (5)
C2—C3—C4—C5	−0.5 (4)	C10—C11—C12—C13	−0.3 (5)
C3—C4—C5—O1	0.2 (4)	C11—C12—C13—O4	172.5 (3)
C3—C4—C5—I1	−174.7 (3)	C11—C12—C13—O5	−6.8 (5)

### (II) N-[(5-Iodofuran-2-yl)methyl]-2-methylpropan-2-aminium (2Z)-3-carboxyprop-2-enoate Hydrogen-bond geometry (Å, º)

**Table:**

*D*—H···*A*	*D*—H	H···*A*	*D*···*A*	*D*—H···*A*
O5—H5*O*···O3	1.18 (5)	1.25 (5)	2.425 (3)	172 (4)
N1—H1*A*···O2	0.88 (4)	1.97 (4)	2.828 (3)	167 (3)
N1—H1*B*···O4^i^	0.88 (4)	1.92 (4)	2.792 (4)	172 (3)

Symmetry code: (i) *x*, *y*, *z*+1.
